# Severe Typhoid Fever Complicated by Superior Mesenteric and Splenic Vein Thrombosis

**DOI:** 10.3390/idr15040038

**Published:** 2023-07-08

**Authors:** Piero Veronese, Marco Pappalardo, Valentina Maffini, Monica Rubini, Alessandra Giacometti, Maria Beatrice Ruozi, Simone Cella, Icilio Dodi

**Affiliations:** 1 Pediatric Infectious Disease, Children’s Hospital of Parma, 43126 Parma, Italy; 2 Pediatric Radiology, Institute of Radiology, University of Parma, 43126 Parma, Italy

**Keywords:** enteric fever, *Salmonella typhi*, thrombosis

## Abstract

Typhoid fever (Typhoid or enteric fever) is still the most common bacterial bloodstream infection worldwide, caused by *Salmonella typhi*. The transmission route is indirect through passive vehicles such as contaminated water or food. Main clinical findings are a fever lasting more than three days, abdominal symptoms, leukocytosis, and anemia. Typhoid can cause a wide range of multi-organ complications. We report a particularly severe form of this infection complicated by superior mesenteric vein and splenic vein thrombosis, an extremely uncommon manifestation.

## 1. Introduction

Typhoid, also known as typhoid and paratyphoid fever collectively referred to as enteric fever, is an invasive infection caused by human host-restricted organism *Salmonella enterica* serovars *typhi* and *paratyphi* [1].

Enteric fever is an important cause of morbidity and mortality mostly in low- and middle-income countries with poor water supply and sanitation, especially Asia and sub-Saharan Africa [2,3]. As of 2019, an estimated 9 × 10^6^ infections and 110 × 10^3^ deaths occur every year worldwide [4], with higher fatality among children and older adults and among those living in low-income countries [5].

The clinical features are highly heterogeneous, ranging from mild symptoms to multiple organ failure; a typical feature is fever with chills, headache, abdominal pain (both diarrhea and constipation are possibilities), myalgias, cough, anorexia, and nausea.

Diagnosis can be difficult on a clinical basis: nonspecific symptoms may mimic other co-endemic gastrointestinal and febrile infections, such as malaria, dengue, leptospirosis, and brucellosis [6].

Following the resolution of the disease, approximately 1–4% of patients progress to the carrier state, excreting the bacilli for more than 1 year [7].

Laboratory findings are also nonspecific, including leukocytosis and mild anemia.

Bacterial cultures and the Widal test are the most common diagnostic tests.

Bone marrow culture has the highest sensitivity, but is difficult to obtain, invasive and is impractical for routine use, whereas other samples show lower sensitivity [8].

The Widal test, a serological test that detects agglutinating anti-bodies against O and H antigens, is a low-cost, simple, and quick-to-execute test but may be difficult to read due to its low sensitivity and specificity, especially in the early stages and in chronic carriers [9,10].

The treatment of typhoid fever normally consists of antibiotics; antimicrobial resistance has become a major threat to the treatment of typhoid with increasing levels of treatment failure due to multidrug resistance (MDR) strains. MDR *S. Typhi* is now considered endemic in many endemic areas [11].

Pylephlebitis represents an uncommon complication of severe abdominal infections and is defined as suppurative phlebitis of the portal vein or its branches [12]; any part of the portal venous system can be affected.

In the systematic review by Jevtic et al. superior mesenteric vein (SMV) is involved in 40% of cases, splenic vein in 12.6% [13]. The most common imaging modalities to diagnose patients with pylephlebitis are abdominal ultrasound (US), computed tomography (CT) and magnetic resonance imaging (MRI) [14].

## 2. Case Description

We describe a case report of a 17-year-old girl from Northern Italy, who was admitted to our Pediatric Infectious Diseases ward because of multi-organ involvement due to enteric fever.

Our patient was first evaluated at the emergency department of another district for abdominal pain, fever, and diarrhea without blood or mucus. These symptoms started about a week after the intake of raw fish (sushi).

Blood exams were performed at that time and showed a normal red (RBC) and white (WBC) blood cells count with elevated C-reactive protein values (CRP 123 mg/L); in addition, the thickening of the wall of intestinal loops (small intestine, along with cecum, ascending and transverse colon) was found at the ultrasound imaging of the abdomen. Microbiological tests on stools were negative, and blood cultures were not performed at that time. The patient was discharged with antimicrobial therapy: Azithromycin 500 mg once a day for 6 consecutive days.

**Figure 1:**
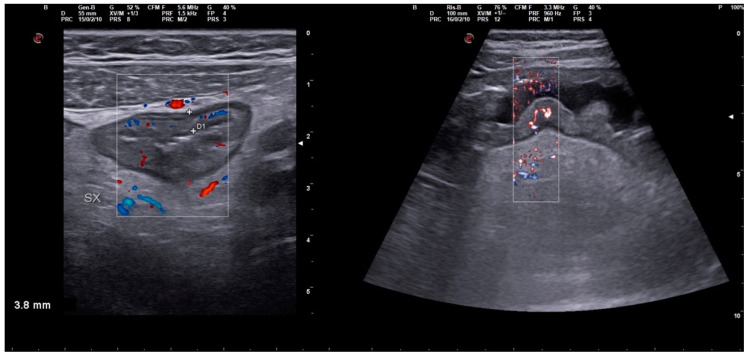
Cross-sectional and longitudinal US image of intestinal loop wall thickening.

**Figure 2:**
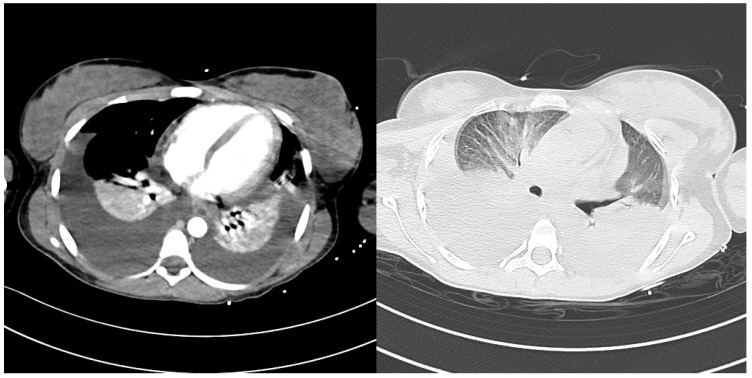
CT image of bilateral pleural effusion.

**Figure 3:**
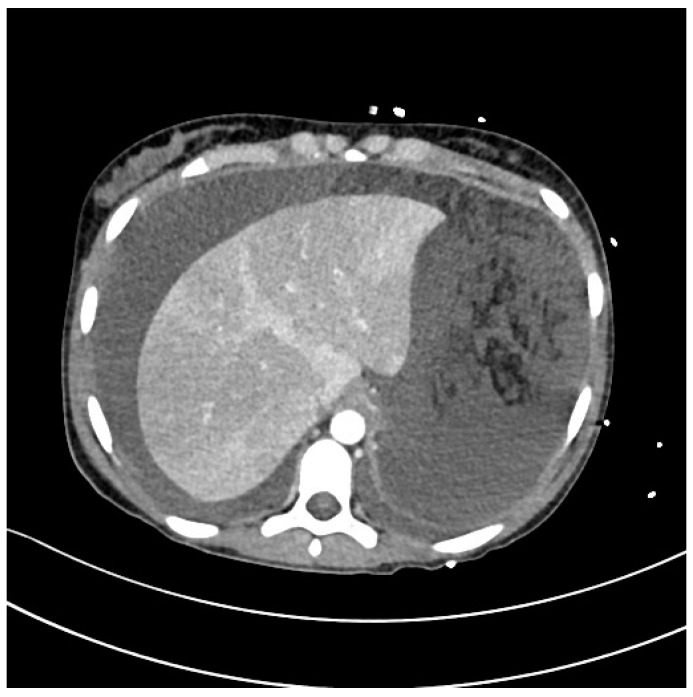
CT scan of bilateral pleural and ascitic effusions and impaired liver density.

**Figure 4:**
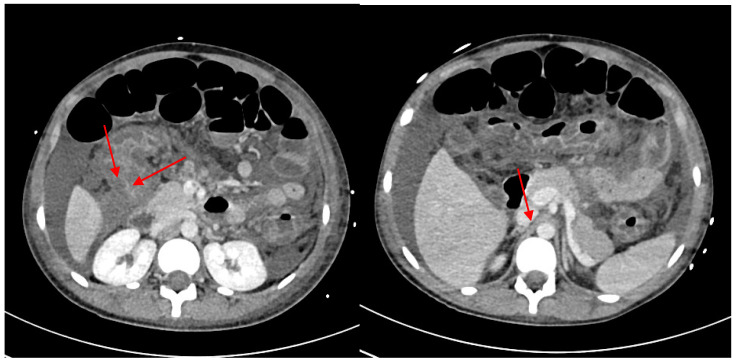
CT scan of superior mesenteric vein and splenic vein thrombosis.

Symptoms improved at first, but after 6 days the patient re-presented at the same facility for the sudden worsening of gastrointestinal symptoms, such as muco-hematic diarrhea (>15 times/day) without febrile state. Blood tests were promptly performed, showing marked neutrophilic leukocytosis (WBC 29,260/μL), low platelet count (Platelet 35,000/μL), mild anemia (Hemoglobin 9.3 g/dL), and elevated C-reactive protein (102 mg/L), in addition to hyponatremia (Sodium 122 mEq/L) and hypoalbuminemia (2.3 g/dL). An abdomen CT scan was also performed and revealed a marked thickening of the transverse and descending colon associated with significant ascitic effusion; focal filling defects of the superior mesenteric vein and a suspected thin thrombotic apposition of the splenic vein were revealed after administration of contrast enhancement. Both ultrasound and CT scan findings are shown in Figure 1, Figure 2, Figure 3 and Figure 4. Figure 1 shows a cross-sectional and longitudinal US image of intestinal loop wall thickening.

From a therapeutic point of view, total parenteral nutrition, albumin supplementation, and empirical broad-spectrum antibiotic therapy with Piperacillin-Tazobactam was undertaken.

Despite therapy, the patient presented an aggravating and sudden worsening of clinical conditions with several muco-hematic diarrhea and respiratory distress; because of the clinical impairment, the patient was transferred to the intensive care unit (ICU) of our hospital.

The onset of marked respiratory distress required a chest CT, which showed bilateral pleural effusion complicated with atelectasis; pleural drains were placed and ventilation by High Flow Nasal Cannula (HFNC) was undertaken.

CT scans with bilateral pleural and ascitic effusions are showed in Figure 2 and Figure 3.

Antimicrobial therapy was implemented with the addition of Metronidazole and Albendazole. Albumin supplementation and total parenteral nutrition were continued.

Cardiological evaluation showed a mild dilatation of coronary arteries with normal morphological and functional parameters. In addition, two blood transfusions were performed for severe anemia (hemoglobin 6.2 g/dL).

Cultures and virus detection on stools were negative; blood culture isolated *Staphylococcus epidermidis*. Ascites and pleural fluid sample cultures were performed but had a negative result. Specific antibodies for *Brucella*, *Epstein-Barr virus* and *Coxsackievirus* were also performed and found to be negative.

After 3 days in the ICU and improvement of general conditions, the patient was transferred to our pediatric department. In our ward, she continued to present diarrheal evacuations albeit in smaller amounts of three to four episodes per day, always lying in the muco-hematic aspect. She never presented fever and general conditions continued to improve. Blood tests were repeated daily and were found to be steadily improving; hemoglobin reached values of 7.6 g/dL with an excellent increase in the reticulocyte count, and no further blood transfusions were needed.

Radiological follow-up with ultrasound imaging of the chest and the abdomen was performed showing complete resolution of the pleural and ascitic effusion.

Careful evaluation of the splenic and portal vein branches was also performed, showing thrombophlebitis at the origin of the superior mesenteric vein and splenic vein; anti-thrombotic therapy with Acetylsalicylic Acid (ASA) 100 mg/day was then undertaken.

From a diagnostic point of view, the result of the Widal test performed at the facility where the girl was previously admitted was reported: *S. paratyphi* A antigen positive 1:160, *S. paratyphi* B antigen H positive 1:160, *S. typhi* antigen O positive 1:640. Clinical history associated with the results of a serological test allowed for the diagnosis of typhoid fever.

At clinical examinations after discharge, the patient gradually but progressively improved her general and nutritional condition. She presented diarrhea for ten days after discharge, and for thirty days with appreciable blood marks.

The echographic checkup performed twenty-one days after discharge was normal and color-doppler examinations of spleno-portal vessels showed a stepwise reduction of the thrombosis and subsequent mild portal hypertension, until complete resolution thirty-five days after discharge.

Antibiotic therapy lasted 14 days, ASA was discontinued 30 days after discharge.

## 3. Discussion

Typhoid (or typhoid fever) is an infectious and systemic disease caused by *Salmonella enterica serovar typhi*, less frequently by *serovar paratyphi A* [15], a human host-restricted organism [16]. The global burden of typhoid fever stands at 9 million cases and 110,000 deaths annually in 2020, mostly in South Asia, Southeast Asia, and sub-Saharan Africa [17,18].

*Salmonella typhi* transmission is considered to be largely indirect through passive vehicles such as contaminated water or food [19]. While *Salmonella* may survive for extended periods on vehicles, multiplication in water and food is uncommon. In high-income countries, most cases are due to return travelers or the intake of raw or undercooked food. The incubation period of the disease is 7–14 days (range 3–30) for *S. enterica serovar typhi*, and 1–10 days for *serovar paratyphi* [20].

The intake of raw fish 7 days before the onset of symptoms was an important element in suspecting enteric fever; other gastrointestinal and febrile bacterial and parasitic infections (e.g., *Anisakis species*) have been hypothesized.

Fever represents the main and earliest clinical feature of enteric fever [7] and other symptoms are heterogeneous and aspecific; typhoid mostly manifests as a gastrointestinal illness, occasionally with blood diarrhea, despite the fact that constipation may be present in the early stages. Other common symptoms are influenza-like myalgias, dull headache, malaise, anorexia, and dry cough [11]. A wide range of complications has been described: bowel perforation (typically at ileum, rarely colon), hepatitis, cholecystitis, pneumonia, myocarditis, acute kidney injury, anemia, meningitis, deep-seated abscess, and hemodynamic shock [21,22].

Febrile state along with gastrointestinal symptoms, initially diarrhea with subsequent appearance of blood trails, were the first clinical manifestations in our patient.

We witnessed a very severe form of this infection; in fact, after the first week with a febrile state, a wide spectrum of symptoms appeared leading to a multi-systemic involvement. From the hematologic point of view, severe anemia and thrombocytopenia required two blood transfusions; respiratory involvement with important pleural effusion necessitated HFNC oxygen support and pleural drains were located. Important ascitic effusion with abdominal colic thickening was also noted. Cardiac involvement was mild and did not lead to hemodynamic shock.

On blood tests, most patients have a total white blood cell count that is within the normal range and leukocytosis may suggest intestinal perforation [23]. Anemia with mean corpuscular hemoglobin, mild thrombocytopenia, and an increased erythrocyte sedimentation rate are common; there may be laboratory markers of a disseminated intravascular coagulation [24]. On laboratory examination, the appearance of significant neutrophilic leukocytosis associated with anemia and thrombocytopenia strengthened our suspicion of typhoid fever.

CT scan with contrast-enhancement and ultrasonography of the abdomen excluded intestinal perforation but enhanced another type of complication such as signs of thrombosis of the portal venous system (superior mesenteric vein, splenic vein) resulting in mild portal hypertension. Thrombophlebitis due to *Salmonella* spp. infection is a very uncommon event: case report of thrombophlebitis affecting cerebral district because of central nervous system involvement is reported [25].

Thrombophlebitis in the portal vein branches is probably due to a suppurative spread of the infection (pylephlebitis). Pylephlebitis occurs by the spread of bacteria through small vessels to vein that drain into the portal vein system [12]. The source of infection is typically intra-abdominal and *Escherichia coli* is the most common pathogen isolated in blood cultures [26]; other isolated bacteria are *Bacterioides* spp., *Streptococcus* spp., and *Fusobacterium* spp. [27,28]. While diverticulitis is the most common associated infection in adult patients, in the pediatric population, pylephlebitis can be associated with umbilical vein catheterization, liver abscess, appendicitis, and enteritis.

The most common reported symptom is fever and CT of the abdomen is considered to be the best exam to make a diagnosis [14].

The treatment of pylephlebitis consists of board-spectrum antibiotics [29], but there is no uniform recommendation regarding the use of anticoagulation, although it can decrease complications of chronic portal hypertension [12,29].

In our case the presence of severe infection and inflammation of the abdomen likely lead to suppurative vein thrombosis. It was described by both the CT and US of the abdomen.

Culture examination from specimens, usually blood and bone marrow, represents the gold standard diagnostic test and provides an isolate for antimicrobial susceptibility testing and molecular characterization. Blood culture is positive in up to 80% of cases. *Salmonella enterica* can be isolated in up to 30% of patients from feces and in less than 1% from urine samples; the number of organisms recoverable from feces increases in untreated illness [30]. Nucleic acid amplification tests, including conventional PCR and real-time PCR, have been developed [31].

The Widal test measures agglutinating antibodies against the LPS (O) and flagellar (H) antigens of *Salmonella enterica* (*serovar typhi-paratyphi*). Serology represents a simple and inexpensive test to perform but may be falsely positive in vaccinated individuals or chronic carriers; a single positive Widal test result in an unvaccinated or unexposed child may have some diagnostic relevance, especially in non-endemic areas. On the other hand, a positive test result in endemic countries is not helpful for diagnosis because of repeated exposures to *Salmonella typhi* and the higher incidence of carrier people, excreting the bacilli [32,33].

In our case, culture specimens on stool and blood failed to isolate *Salmonella*; blood culture isolated *Staphylococcus epidermidis*: this finding was considered a result of contamination taking into account clinical features and symptoms. The culture of bone marrow sample and the test with higher sensitivity, was not performed. In contrast, serological investigation (Widal test) was strongly suggestive of an ongoing infection (*S. typhi* antigen O positive 1:640). Despite the low specificity of serological test, the association of the history of exposure in a patient living in a non-endemic area, clinical features, and laboratory findings strengthened its diagnostic value; these features allow us to move definitively toward the diagnosis of enteric fever.

Antimicrobial therapy is needed to resolve infection, instead of general supportive care, along with close monitoring for disease complications. Multidrug-resistant strains are endemic in many countries; resistance to traditional first-line antimicrobials ampicillin, chloramphenicol and trimethoprim-sulfamethoxazole is common and is caused by resistance determinants localized on plasmids.

Board-spectrum antimicrobial therapy Piperacillin-Tazobactam and Metronidazole was promptly started and has been effective [34]. Albendazole was undertaken in suspicion of Anisakis infection.

Finally, regarding antimicrobial therapy, early treatment with macrolide probably mitigated symptoms during the initial febrile phase; however, it was not enough to achieve the complete eradication of the infection [35].

Because of the significant bowel involvement, it was necessary to undertake total parenteral nutrition.

The need for anticoagulant or antithrombotic therapy has been a topic of management; ASA was started and discontinued when an US exam of the abdomen showed no more existance of phlebitis, 30 days after discharge.

## 4. Conclusions

Typhoid fever is still the most common bacterial bloodstream infection worldwide, especially in low-income countries. In non-endemic high-income countries it should always be considered in case of febrile illness, focusing on the history of potentially contaminated food and raw or undercooked food consumption or recent travel in endemic areas. A fever lasting more than three days, abdominal symptoms, leukocytosis and anemia must always be suspected as symptoms of typhoid fever.

The clinical course is heterogeneous and can be particularly severe with systemic involvement and multiorgan complications such as enteropathy, polyserositis, cardiac involvement, and severe anemia.

Thrombophlebitis is rare but feasible in the affected districts as a suppurative complication (pylephlebitis); in the case of severe intestinal involvement, infection can spread through small vessels and lead to venous obstruction of the portal vein system up to pre-hepatic portal hypertension; although uncommon, thrombophlebitis must be carefully evaluated by the physician. Imaging of the abdomen usually demonstrates pylephlebitis.

The serological test (Widal test) is weighed down by low specificity when compared to culture or molecular biology tests; however, in non-endemic countries, where the presence of chronic carriers is low, it can be useful for diagnostic purposes, especially when a history of exposition, clinical, and laboratory findings are suitable.

Board-spectrum antimicrobial therapy is required to treat infection, but isolated *Salmonella* can result in multiple antibiotic resistance, especially in endemic areas.

The treatment of suppurative thrombophlebitis also needs board-spectrum antibiotics, while there is no uniform recommendation regarding the use of anticoagulation.

## Data Availability

Data sharing not applicable.
